# Spinal Versus General Anesthesia for Acute Kidney Injury and Transfusion in One-Week-Staged Bilateral Total Knee Arthroplasty

**DOI:** 10.3390/jcm15134937

**Published:** 2026-06-25

**Authors:** Jaemin Lee, Jun Suh Moon, Doo Sup Kim

**Affiliations:** Department of Orthopedic Surgery, Wonju College of Medicine, Yonsei University, Wonju 26426, Republic of Korea; megatal12@yonsei.ac.kr (J.L.); ilovejsm1@gmail.com (J.S.M.)

**Keywords:** total knee arthroplasty, spinal anesthesia, general anesthesia, acute kidney injury, blood transfusion, postoperative complications, intention-to-treat analysis

## Abstract

**Background/Objectives:** Evidence on spinal versus general anesthesia in unilateral total knee arthroplasty (TKA) may not extend to one-week-staged bilateral surgery, where older patients receive two anesthetics in a short interval and intra-operative spinal-to-general conversion is common but rarely reported transparently. We compared peri-operative acute kidney injury (AKI) and transfusion between strategies in this setting. **Methods:** We retrospectively analyzed 207 patients (414 surgeries) undergoing one-week-staged bilateral primary TKA at one center. Co-primary endpoints were creatinine-based AKI (patient level) and packed-red-blood-cell transfusion (surgery level). Because 42 general-anesthesia-classified surgeries had an attempted spinal injection, the primary analysis used the initial anesthetic plan (an intention-to-treat analogue), reclassifying these as spinal, with as-treated classification as a sensitivity analysis; AKI was modeled at the patient level (any general anesthesia versus spinal–spinal) and transfusion per surgery. **Results:** Median age was 75 years and 82.6% were female; AKI affected 74 of 207 patients (35.7%) and transfusion 185 of 414 surgeries (44.7%). The adjusted any-general-anesthesia versus spinal–spinal estimate was not statistically significant and opposite the spinal-protective hypothesis (adjusted odds ratio 0.49, 95% confidence interval 0.23–1.01, *p* = 0.054), and no pre-specified sensitivity scenario survived Benjamini–Hochberg correction. Transfusion did not differ between strategies; among secondary endpoints, length of stay, hemoglobin drop, peak C-reactive protein, and intra-operative hypotension likewise showed no significant difference after multiplicity correction. **Conclusions:** These hypothesis-generating findings do not support changing anesthetic practice; the choice should remain individualized. Approximately 12% of attempted spinal anesthetics converted intra-operatively to general anesthesia—a record-based observation, not a validated failure rate.

## 1. Introduction

End-stage knee osteoarthritis disables many older adults, and the demand for total knee arthroplasty (TKA) is projected to rise substantially over the coming decades [1]. TKA carries important peri-operative risks, including bleeding that may require transfusion and acute kidney injury (AKI) [2,3]. Patients with bilateral disease often progress to bilateral TKA (BTKA), for which optimal scheduling remains debated [4]: simultaneous BTKA consolidates both knees into a single anesthetic and admission but carries higher rates of major complications, including AKI and transfusion [5,6,7,8], though chronic-opioid use is similar between approaches [9], and staged BTKA within a single admission—commonly at a one-week interval—has gained traction, particularly in Korean and Asian centers [10,11], prompting a recent international consensus on the optimal interval [12]. Anesthesia choice has likewise drawn scrutiny: in unilateral TKA, large-database and single-institution analyses and a meta-analysis of more than two million arthroplasty cases consistently favor spinal anesthesia (SA) over general anesthesia (GA) for length of stay, readmission, opioid use, and transfusion [13,14,15,16,17,18,19,20]. The picture for AKI is less consistent: Korean analyses report lower acute renal failure under regional anesthesia [21] or lower mortality without a renal-failure difference [22], a single-center study of 2987 TKAs found only a non-significant trend favoring SA [23], a machine-learning analysis flagged GA as an AKI risk factor [24], Veterans Affairs data linked neuraxial anesthesia to lower renal complication risk [25], and a four-arm randomized trial found no significant recovery advantage of spinal over general anesthesia [26].

Most prior evidence stems from unilateral TKA or simultaneous BTKA under a single anesthetic, and data on anesthesia choice in staged BTKA—two anesthetic exposures within a short interval—remain sparse despite growing adoption [27,28]. This gap matters clinically: bilateral surgery amplifies peri-operative AKI risk relative to unilateral procedures [5,6,29], and the renal and hemodynamic stress of the first procedure may not have resolved when the second anesthetic is delivered about one week later, so the second-surgery choice is made in an already physiologically primed patient; whether the interval itself modifies outcomes remains debated, with one-week versus delayed staging showing no difference in 90-day complications but higher transfusion risk in the short-staged group [27]. Another blind spot is that a non-trivial share of attempted spinal anesthetics in older adults converts intra-operatively to GA, yet observational studies rarely report whether such conversions are handled under intention-to-treat (ITT) or as-treated (AT) frameworks—a choice that can shift inferences about either technique’s protective effect.

In this single-institution cohort of older Korean adults undergoing one-week-staged primary BTKA, we compared the adjusted risk of peri-operative AKI, defined by Kidney Disease: Improving Global Outcomes (KDIGO) creatinine-based criteria, and of transfusion between SA and GA, both per surgery and at the patient level, with length of stay, intra-operative hypotension, peri-operative hemoglobin drop, and peak post-operative C-reactive protein as secondary endpoints. The pre-specified hypothesis, recorded in the institutional analysis plan before model fitting, followed the unilateral-TKA literature in expecting lower AKI and transfusion risk with SA than GA—neuraxial anesthesia having been associated in that literature with reduced blood loss and transfusion through lower intra-operative venous pressure and attenuation of the surgical stress response. To address spinal-anesthesia failure with intra-operative conversion to GA, we pre-specified an ITT primary and an AT sensitivity analysis, and we also examined whether the anesthesia pattern across the two surgeries (SA–SA, GA–GA, SA–GA, GA–SA) carried independent prognostic information. By treating AKI and transfusion as dual co-primary endpoints and contrasting ITT with as-treated handling of conversion, this study adds evidence to a sparsely studied population and makes explicit how conversion handling and creatinine-baseline choice influence AKI inference in staged-bilateral arthroplasty.

## 2. Materials and Methods

### 2.1. Study Design and Setting

This single-center retrospective cohort study followed the Declaration of Helsinki and is reported in accordance with the Strengthening the Reporting of Observational Studies in Epidemiology (STROBE) statement [30]. We analyzed adults who underwent staged bilateral primary TKA at our institution between January 2020 and April 2026. The Institutional Review Board of Wonju Severance Christian Hospital approved the study (IRB No. CR326057; approved 19 May 2026) and waived written informed consent given the retrospective design and use of de-identified electronic medical record (EMR) data. The study period denotes the calendar span of the surgeries retrospectively reviewed; ethical approval was granted before any patient records were accessed, and all data were de-identified before analysis.

### 2.2. Participants

**Figure 1:**
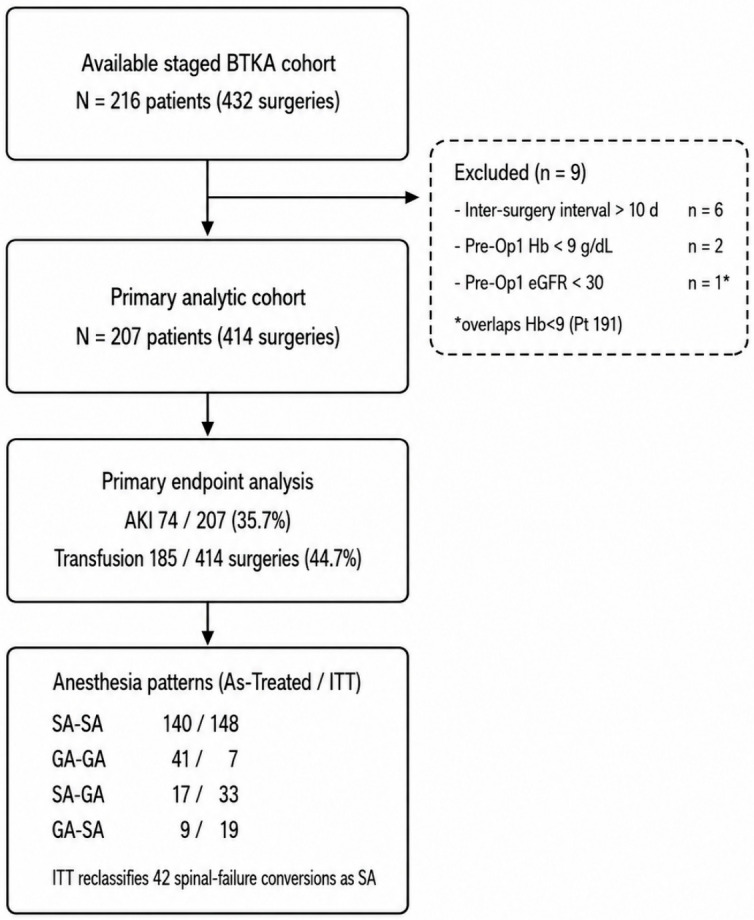
Patient flow. Screening, exclusion, and the final analytic cohort (207 patients, 414 surgeries). The 9 unique exclusions comprised 6 patients with a 14-day inter-surgery interval, 2 with pre-Op1 hemoglobin below 9.0 g/dL, and 2 with pre-Op1 eGFR below 30 mL/min/1.73 m^2^; one patient met both the hemoglobin and eGFR criteria and is counted once under hemoglobin, so the reason-specific counts shown in the diagram (6, 2, and 1) sum to 9. Anesthesia-pattern distribution under as-treated and intention-to-treat classifications is shown.

Patients were eligible if aged at least 55 years, underwent staged bilateral primary TKA during a single admission, and had an inter-surgery interval of 10 days or fewer. We excluded patients with an interval exceeding 10 days, a pre-operative hemoglobin (Hb) before the first surgery below 9.0 g/dL, or a pre-operative estimated glomerular filtration rate (eGFR) below 30 mL/min/1.73 m^2^ (KDIGO chronic kidney disease stage 4–5); patients on chronic dialysis or with end-stage renal disease were excluded a priori, because creatinine-based KDIGO staging is undefined there. Of 216 patients screened, 9 unique patients were excluded—6 with a 14-day interval reflecting the next-scheduled-week protocol, 2 with pre-Op1 Hb below 9.0 g/dL, and 2 with pre-Op1 eGFR below 30 (one met both criteria, so raw counts sum to 10 but the net unique exclusion is 9)—leaving 207 patients corresponding to 414 surgeries (Figure 1).

### 2.3. Anesthesia and Surgical Protocols

For each patient, the same attending anesthesiologist managed both staged surgeries and selected the anesthesia type for each. SA used hyperbaric bupivacaine (typically 9–12 mg) at the L3–L4 or L4–L5 interspace, with intra-operative sedation in all spinal cases; GA was induced with intravenous propofol and maintained with sevoflurane or desflurane, supplemented with opioids and neuromuscular blockade. Intra-operative hemodynamic management followed routine institutional practice rather than a fixed mean-blood-pressure target, with intravenous ephedrine or phenylephrine at the anesthesiologist’s discretion. Post-operative analgesia was standardized across both anesthesia groups: intravenous fentanyl-based patient-controlled analgesia plus multimodal adjuncts (acetaminophen and, when not contraindicated, a non-steroidal anti-inflammatory drug). Forty-two surgeries (approximately 12% of all attempted spinal anesthetics) originally classified as GA documented an attempted spinal injection at a bona fide spinal dose (9–12 mg), interpreted as spinal-anesthesia failure converted to GA; these were identified by an automated text-and-dosing query, after which the operating anesthesiologist reviewed the records and confirmed all 42 as genuine intra-operative spinal-anesthesia failures rather than intentional combined spinal–general techniques, and because the same anesthesiologist managed both surgeries, conversion decisions were applied consistently within patients. Experienced arthroplasty surgeons operated using a pneumatic tourniquet (250–300 mmHg, released after full cement curing), an antero-medial parapatellar approach, cemented posterior-stabilized implants, and routine wound drainage. Tranexamic acid was not administered as part of routine protocol. Packed red blood cell (PRBC) transfusion was given for a hemoglobin below 8 g/dL or symptomatic anemia, and venous thromboembolism prophylaxis comprised intermittent pneumatic compression with early mobilization.

### 2.4. Variables

The primary exposure was the anesthesia technique for each surgery (SA versus GA), classified under two frameworks: the primary initial-anesthetic-plan (ITT) framework reclassified spinal-to-GA conversions as SA, on the rationale that the intended exposure carries the most direct clinical interpretation, whereas the as-treated framework classified surgeries by the technique actually used. Because exposure was clinically rather than randomly assigned, “intention-to-treat” here denotes the initially planned anesthetic rather than the classical randomized estimand. At the patient level, anesthesia patterns across the two surgeries were SA–SA, GA–GA, SA–GA, or GA–SA.

The co-primary outcomes were peri-operative AKI and transfusion. AKI followed the KDIGO creatinine-based criteria [31] against the single pre-Op1 baseline: Stage 1 or higher corresponded to a peak post-operative creatinine at least 1.5 times the baseline or at least 0.3 mg/dL above it, across post-operative measurement windows labeled post-operative day (POD) 0 through 5 for both surgeries; the urine-output criterion was not applied, as hourly urine output was not reliably available. Patient-level binary AKI (Stage 1 or higher in either surgery) was primary, with per-surgery AKI also analyzed. Transfusion was PRBC transfusion given intra-operatively or between POD0 and discharge, modeled as binary occurrence and units per surgery; each transfusion was attributed in the source records to the individual surgery during or after which it was administered (separate per-surgery fields for Op1 and Op2). Because a transfusion around the second surgery may partly reflect cumulative blood loss carried over from the first surgery only about one week earlier, the surgery-level models accommodated this within-patient carryover and correlation through a patient random intercept (binary occurrence) or patient-clustered generalized estimating equations (unit counts), together with a surgery-number term (Section 2.6). Secondary outcomes were length of stay (LOS; total hospital days from the first surgery to discharge), intra-operative hypotension, quantified per surgery as the number and cumulative duration of discrete episodes with mean blood pressure [MBP] below 65 mmHg—an episode-based metric used in place of a single averaged value, so that brief but clinically important hypotensive periods were not masked by averaging, peri-operative Hb drop (pre-Op1 Hb minus the POD2 nadir for each surgery; because this uses the common pre-Op1 baseline, the second-surgery value also reflects cumulative decline carried over from the first surgery, so the within-surgery local drop—each surgery’s own immediate pre-operative Hb minus its nadir—is reported separately in Supplementary Table S6), and peak post-operative C-reactive protein (CRP). Composite complications (hematoma, surgical site infection [SSI], deep vein thrombosis [DVT], pulmonary embolism [PE], or reoperation) were reported descriptively given the very low event rate.

A priori covariates were age, sex, body mass index (BMI), American Society of Anesthesiologists (ASA) physical status (3 or higher), hypertension, diabetes mellitus, cardiovascular disease, chronic kidney disease (a documented EMR comorbidity, not derived from eGFR), antiplatelet or anticoagulant use, baseline pre-Op1 creatinine and/or hemoglobin as relevant to each endpoint, tourniquet time, and operation time; patient-level models used across-surgery averages of tourniquet and operation times, and surgery-level models used per-surgery values plus surgery number. With 74 events and 14 coefficients (events-per-variable ≈ 5), the patient-level AKI estimates are interpreted as exploratory.

### 2.5. Data Sources and Measurement

Demographics, comorbidities, surgical and anesthesia records, transfusion records, and complications were extracted from the EMR and verified by direct chart review—except that spinal-to-general conversion status was first identified by the automated text-and-dosing query described above and then confirmed by the operating anesthesiologist rather than by an independent blinded per-case audit—and laboratory data (creatinine, electrolytes, hemoglobin, CRP, albumin) were integrated by a per-test, window-based closest-measurement algorithm. The pre-Op1 baseline was the closest creatinine within 180 days before Op1, post-operative creatinine windows spanned POD0–POD5 (POD2 and POD5 defined as days +2–3 and +4–7) for both surgeries, and post-Op2 CRP was the first outpatient measurement within 7–180 days after Op2 (outpatient CRP-panel values taking precedence over duplicates). Serum creatinine was available at every protocol-defined post-operative window for nearly all patients (Supplementary Table S3). We calculated eGFR by the race-free Chronic Kidney Disease Epidemiology Collaboration (CKD-EPI) 2021 equation. Because a single pre-Op1 baseline can conflate unresolved post-Op1 elevation with new post-Op2 injury, a post hoc analysis redefined the Op2 baseline as the latest pre-Op2 creatinine. Two implausible laboratory values were excluded as confirmed data-entry errors, and 13 patients without post-Op2 CRP (12 within the cohort) were imputed with the cohort median under an explicit flag. No other variables were imputed; regression models used complete-case data.

We sought to address potential bias through a complete consecutive series (selection), multivariable adjustment with propensity-score-matched and weighted analyses (confounding by indication), the dual ITT/AT analysis (exposure misclassification), laboratory-test-date creatinine windows and the Op2-specific baseline (AKI ascertainment), and patient random intercepts in the mixed-effects models plus patient-clustered generalized estimating equations (GEE) for count outcomes (within-patient correlation).

### 2.6. Statistical Analysis

Descriptive statistics were mean ± standard deviation (normal), median [interquartile range, IQR] (non-normal), or n (%); normality used Shapiro–Wilk (n < 50 per group) or D’Agostino–Pearson. Univariable comparisons used the Student’s *t*-test or Mann–Whitney U test and the χ^2^ or Fisher exact test; the sparse-celled four-group comparisons in Table 1 are descriptive only, and the surgery-level Table 2 is presented descriptively without inferential *p*-values, because its rows duplicate each patient’s invariant characteristics across both surgeries (violating independence), so all comparative inference is reported in Table 3. For the primary AKI endpoint, patient-level logistic regression compared any GA exposure (any pattern other than SA–SA) with SA–SA, and a four-pattern model (reference SA–SA) was fit as a secondary, exploratory analysis given that the GA–GA cell held only seven patients. Surgery-level binary AKI and transfusion occurrence used mixed-effects logistic regression with a patient random intercept, reporting adjusted odds ratios (aOR) with 95% confidence intervals (CI); transfusion units used negative binomial (NB) GEE with robust standard errors (over-dispersion, variance/mean = 1.13), and patient-level total PRBC units used NB regression; because transfusion occurrence was modeled separately as a binary outcome, the large fraction of zero-unit surgeries was captured by that first-stage occurrence model (a hurdle-type two-part separation), so a combined zero-inflated or hurdle count model was not additionally required, and the occurrence and unit models yielded concordant null results. Secondary endpoints used linear regression (LOS and patient-level peak log-CRP), linear mixed-effects models (Hb drop), and NB GEE (hypotension counts), with the LOS model additionally adjusted for the inter-surgery interval and the peak-CRP model for baseline albumin. To verify that the borderline primary estimate was not an artifact of the events-per-variable ratio, we re-estimated the any-GA and four-pattern AKI models with Firth penalized-likelihood regression and refit the any-GA model using the propensity score as a single covariate and a reduced five-covariate specification (Supplementary Table S4), retaining the maximum-likelihood estimate as primary.

**Table 1:** Baseline characteristics and outcomes by anesthesia pattern (intention-to-treat).

Variable	Overall (n = 207)	SA–SA (n = 148)	GA–GA (n = 7)	SA–GA (n = 33)	GA–SA (n = 19)	Test	*p*
**Demographics**							
Age, y	75.0 [70.0, 80.0]	76.0 [70.0, 80.0]	77.0 [72.5, 81.0]	73.0 [69.0, 76.0]	72.0 [69.0, 77.0]	Kruskal–Wallis	0.120
BMI, kg/m^2^	26.6 [24.3, 29.1]	26.3 [24.2, 28.5]	27.7 [26.4, 30.4]	26.9 [24.6, 30.0]	28.4 [25.1, 33.3]	Kruskal–Wallis	0.093
ASA score	3.0 [2.0, 3.0]	3.0 [2.0, 3.0]	2.0 [2.0, 3.0]	3.0 [2.0, 3.0]	2.0 [2.0, 3.0]	Kruskal–Wallis	0.227
Inter-surgery interval, days	7.0 [7.0, 7.0]	7.0 [7.0, 7.0]	7.0 [7.0, 7.0]	7.0 [7.0, 7.0]	7.0 [7.0, 7.0]	Kruskal–Wallis	0.646
Male, n (%)	36 (17.4%)	23 (15.5%)	2 (28.6%)	8 (24.2%)	3 (15.8%)	Chi-square	0.557
ASA ≥ 3, n (%)	118 (57.0%)	85 (57.4%)	3 (42.9%)	22 (66.7%)	8 (42.1%)	Chi-square	0.313
**Comorbidities**							
Hypertension, n (%)	166 (80.2%)	116 (78.4%)	7 (100.0%)	27 (81.8%)	16 (84.2%)	Chi-square	0.516
Diabetes, n (%)	73 (35.3%)	54 (36.5%)	2 (28.6%)	12 (36.4%)	5 (26.3%)	Chi-square	0.821
Cardiovascular disease, n (%)	41 (19.8%)	30 (20.3%)	0 (0.0%)	10 (30.3%)	1 (5.3%)	Chi-square	0.087
Chronic kidney disease, n (%)	4 (1.9%)	1 (0.7%)	1 (14.3%)	2 (6.1%)	0 (0.0%)	Chi-square	0.017
Antiplatelet, n (%)	56 (27.1%)	40 (27.0%)	3 (42.9%)	10 (30.3%)	3 (15.8%)	Chi-square	0.516
Anticoagulant, n (%)	7 (3.4%)	5 (3.4%)	0 (0.0%)	1 (3.0%)	1 (5.3%)	Chi-square	0.927
**Baseline laboratory**							
Pre-Op1 Hb, g/dL	13.1 [12.4, 13.9]	13.1 [12.4, 13.9]	13.1 [12.6, 13.6]	12.8 [12.4, 14.1]	13.1 [12.1, 13.5]	Kruskal–Wallis	0.741
Pre-Op1 Cr, mg/dL	0.8 [0.6, 0.9]	0.8 [0.7, 0.9]	0.7 [0.6, 0.8]	0.8 [0.6, 1.0]	0.8 [0.6, 1.1]	Kruskal–Wallis	0.786
Pre-Op1 eGFR, mL/min/1.73 m^2^	84.7 [68.8, 92.2]	83.6 [69.9, 92.1]	90.2 [79.0, 94.3]	86.4 [65.7, 92.3]	81.9 [60.5, 95.7]	Kruskal–Wallis	0.832
Pre-Op1 Albumin, g/dL	4.4 [4.3, 4.6]	4.4 [4.3, 4.6]	4.5 [4.5, 4.5]	4.4 [4.2, 4.6]	4.5 [4.3, 4.6]	Kruskal–Wallis	0.264
**Surgical times**							
Op1 tourniquet time, min	55.0 [50.0, 64.5]	55.0 [50.0, 62.2]	55.0 [54.5, 67.5]	58.0 [50.0, 64.0]	58.0 [52.5, 63.5]	Kruskal–Wallis	0.478
Op2 tourniquet time, min	55.0 [50.0, 62.5]	55.0 [50.0, 60.0]	57.0 [50.0, 60.5]	56.0 [50.0, 68.0]	55.0 [50.0, 64.0]	Kruskal–Wallis	0.370
Op1 operation time, min	90.0 [81.0, 100.0]	90.0 [80.0, 100.0]	100.0 [82.5, 110.0]	90.0 [84.0, 97.0]	87.0 [85.0, 95.0]	Kruskal–Wallis	0.717
Op2 operation time, min	90.0 [80.0, 95.0]	85.0 [80.0, 95.0]	90.0 [87.5, 97.5]	90.0 [85.0, 95.0]	90.0 [85.0, 93.0]	Kruskal–Wallis	0.224
Op1 anesthesia time, min	140.0 [130.0, 150.0]	135.0 [130.0, 145.0]	155.0 [150.0, 165.0]	150.0 [135.0, 155.0]	150.0 [145.0, 152.5]	Kruskal–Wallis	**<0.001**
Op2 anesthesia time, min	140.0 [130.0, 150.0]	135.0 [125.0, 140.0]	150.0 [143.0, 160.0]	150.0 [140.0, 155.0]	145.0 [140.0, 152.5]	Kruskal–Wallis	**<0.001**
**Outcomes**							
AKI (KDIGO ≥ 1), n (%)	74 (35.7%)	58 (39.2%)	2 (28.6%)	11 (33.3%)	3 (15.8%)	Chi-square	0.231
Any transfusion (either Op), n (%)	138 (66.7%)	100 (67.6%)	6 (85.7%)	22 (66.7%)	10 (52.6%)	Chi-square	0.410
Total PRBC units	1.0 [0.0, 2.0]	1.0 [0.0, 2.0]	1.0 [1.0, 3.0]	1.0 [0.0, 2.0]	1.0 [0.0, 2.0]	Kruskal–Wallis	0.469
LOS, days	15.0 [14.0, 15.0]	14.0 [14.0, 15.0]	15.0 [14.5, 15.0]	14.0 [14.0, 15.0]	15.0 [14.5, 16.5]	Kruskal–Wallis	0.053
Peak post-op CRP, mg/dL	10.9 [7.7, 15.3]	10.9 [7.5, 14.7]	8.4 [7.7, 11.1]	11.2 [8.8, 16.0]	11.2 [7.8, 17.7]	Kruskal–Wallis	0.545
Any complication, n (%) ^1^	4 (1.9%)	2 (1.4%)	1 (14.3%)	1 (3.0%)	0 (0.0%)	descriptive	n/a

Continuous: median [IQR]; categorical: n (%). Patterns classified under intention-to-treat (the initial anesthetic plan; 42 spinal-failure conversions reassigned to SA). Four-group categorical comparisons used the χ^2^ test; several rows have sparse cells (expected count <5; e.g., chronic kidney disease, minimum expected count 0.14), so these baseline-balance *p*-values are descriptive only and were not used for inference. Bold *p* < 0.05 (continuous comparisons, Kruskal–Wallis). ^1^ Composite complication (hematoma/SSI/DVT/PE/reoperation). With only 4 events distributed across 4 pattern cells (sparsest cell n = 1), inferential testing is unreliable; the row is shown descriptively and *p* is not reported; n/a, not applicable. Abbreviations: SA, spinal anesthesia; GA, general anesthesia; BMI, body mass index; ASA, American Society of Anesthesiologists; Hb, hemoglobin; Cr, creatinine; eGFR, estimated glomerular filtration rate; AKI, acute kidney injury; KDIGO, Kidney Disease: Improving Global Outcomes; PRBC, packed red blood cells; LOS, length of stay; CRP, C-reactive protein; SSI, surgical site infection; DVT, deep vein thrombosis; PE, pulmonary embolism.

**Table 2:** Baseline characteristics and outcomes by per-surgery anesthesia type (intention-to-treat, surgery-level).

Variable	Overall (n = 414)	SA (n = 348)	GA (n = 66)
**Patient characteristics**			
Age, y	74.9 ± 6.8	75.1 ± 6.8	73.8 ± 6.4
BMI, kg/m^2^	26.6 [24.3, 29.1]	26.4 [24.2, 28.9]	27.7 [25.5, 31.2]
ASA score	3.0 [2.0, 3.0]	3.0 [2.0, 3.0]	3.0 [2.0, 3.0]
Male, n (%)	72 (17.4%)	57 (16.4%)	15 (22.7%)
ASA ≥ 3, n (%)	236 (57.0%)	200 (57.5%)	36 (54.5%)
**Comorbidities**			
Hypertension, n (%)	332 (80.2%)	275 (79.0%)	57 (86.4%)
Diabetes, n (%)	146 (35.3%)	125 (35.9%)	21 (31.8%)
Cardiovascular disease, n (%)	82 (19.8%)	71 (20.4%)	11 (16.7%)
Chronic kidney disease, n (%)	8 (1.9%)	4 (1.1%)	4 (6.1%)
Antiplatelet, n (%)	112 (27.1%)	93 (26.7%)	19 (28.8%)
Anticoagulant, n (%)	14 (3.4%)	12 (3.4%)	2 (3.0%)
**Baseline laboratory**			
Pre-Op1 Hb, g/dL	13.2 ± 1.2	13.2 ± 1.2	13.0 ± 1.2
Pre-Op1 Cr, mg/dL	0.8 [0.6, 0.9]	0.8 [0.6, 0.9]	0.8 [0.6, 1.0]
Pre-Op1 eGFR, mL/min/1.73 m^2^	84.7 [68.7, 92.3]	83.7 [69.2, 92.1]	86.8 [67.7, 94.0]
Pre-Op1 Albumin, g/dL	4.4 [4.3, 4.6]	4.4 [4.3, 4.6]	4.5 [4.3, 4.6]
**Intra-operative**			
Tourniquet time, min	55.0 [50.0, 63.0]	55.0 [50.0, 62.0]	56.5 [50.2, 65.8]
Operation time, min	90.0 [80.0, 95.0]	90.0 [80.0, 95.0]	90.0 [85.0, 95.0]
Anesthesia time, min	140.0 [130.0, 150.0]	135.0 [130.0, 145.0]	150.0 [140.0, 155.0]
Intake fluid, mL	950.0 [800.0, 1200.0]	950.0 [800.0, 1200.0]	1075.0 [900.0, 1125.0]
MBP minimum, mmHg	62.0 [58.0, 66.5]	62.0 [58.5, 66.5]	62.0 [58.0, 66.5]
MBP mean, mmHg	82.6 [77.8, 87.8]	82.4 [77.5, 87.8]	85.8 [82.8, 87.3]
**Primary outcomes**			
AKI (KDIGO ≥ 1) per surgery, n (%)	94 (22.7%)	84 (24.1%)	10 (15.2%)
Transfusion per surgery, n (%)	185 (44.7%)	156 (44.8%)	29 (43.9%)
PRBC units per surgery	0.0 [0.0, 1.8]	0.0 [0.0, 1.0]	0.0 [0.0, 2.0]
**Intra-operative events**			
MBP < 65 mmHg events, count	1.0 [0.0, 3.0]	1.0 [0.0, 3.0]	0.5 [0.0, 2.2]
MBP < 65 mmHg duration, min	5.0 [0.0, 15.0]	5.0 [0.0, 15.0]	2.5 [0.0, 11.2]

Surgery-level under intention-to-treat (the initial anesthetic plan; 42 spinal-failure conversions reassigned to SA). Values: mean ± SD (normal), median [IQR] (non-normal), or n (%). This table is descriptive only and reports no inferential *p*-values: the patient-level characteristics (patient characteristics, comorbidities, baseline laboratory) are duplicated across each patient’s two surgeries, which violates the independence assumption of univariable tests, and the surgery-level outcomes are correlated within patients. All comparative inference accounting for within-patient clustering is presented in Table 3. Operation time denotes the recorded surgical duration, whereas anesthesia time denotes the total anesthesia-controlled interval (induction or neuraxial-block placement, intra-operative management, and emergence) and therefore exceeds operation time by the peri-surgical anesthesia phases; the longer anesthesia time under GA reflects these phases rather than a longer operation. The mean and minimum MBP are descriptive summaries of the intra-operative monitoring record; intra-operative hypotension was additionally quantified as the count and cumulative duration of MBP < 65 mmHg episodes (Table 4), so that transient hypotensive periods were not masked by averaging. Abbreviations as in Table 1; MBP, mean blood pressure.

**Table 3:** Adjusted models for primary endpoints.

**(A) Acute Kidney Injury (KDIGO ≥ Stage 1)**				
**Model**	**Exposure**		**aOR (95% CI)**	***p***
**Patient-level logistic, ITT (primary)**	Any GA exposure vs. SA–SA		0.49 (0.23–1.01)	0.054
Patient-level logistic, AT (sensitivity)	Any GA exposure vs. SA–SA		0.61 (0.31–1.22)	0.163
4-pattern (ITT), reference SA–SA	GA–GA pattern		0.60 (0.10–3.76)	0.589
	SA–GA pattern		0.69 (0.29–1.63)	0.393
	GA–SA pattern		**0.21 (0.05–0.82)**	**0.025**
**Surgery-level mixed-effects, ITT**	GA per surgery		0.47 (0.20–1.08)	0.075
Surgery-level mixed-effects, AT (sensitivity)	GA per surgery		0.58 (0.31–1.08)	0.088
**(B) Peri-Operative Transfusion**				
**Model**	**Outcome**	**Exposure**	**Adjusted Effect (95% CI)**	***p***
**Surgery-level mixed-effects logistic, ITT**	Transfusion occurrence	GA per surgery	aOR 0.90 (0.50–1.62)	0.728
Surgery-level mixed-effects logistic, AT	Transfusion occurrence	GA per surgery	aOR 1.13 (0.72–1.77)	0.609
**Surgery-level NB GEE, ITT**	Transfusion units	GA per surgery	aIRR 1.05 (0.76–1.47)	0.760
Surgery-level NB GEE, AT	Transfusion units	GA per surgery	aIRR 1.07 (0.80–1.42)	0.665
Patient-level NB, ITT	Total PRBC units	Any GA exposure	aIRR 0.99 (0.64–1.53)	0.972

All models adjusted for age, sex, BMI, ASA ≥ 3, comorbidities (hypertension, diabetes mellitus, cardiovascular disease, chronic kidney disease), antiplatelet/anticoagulant, tourniquet and operation times, and the outcome-relevant baseline laboratory value (baseline creatinine in panel A [AKI]; baseline hemoglobin in panel B [transfusion]), with surgery-level models additionally adjusted for surgery number. Surgery-level binary models (acute kidney injury, transfusion occurrence) used mixed-effects logistic regression with a patient-level random intercept; surgery-level count models (transfusion units) used negative binomial generalized estimating equations with robust standard errors. Both account for within-patient clustering of the two staged surgeries. Bold *p* < 0.05. Abbreviations: ITT, intention-to-treat (initial anesthetic plan); AT, as-treated; aOR, adjusted odds ratio; aIRR, adjusted incidence rate ratio; NB, negative binomial; GEE, generalized estimating equations. Other abbreviations as in Table 1 and Table 2.

**Table 4:** Secondary endpoints: Adjusted effects of GA exposure (intention-to-treat) with BH-FDR correction.

Endpoint	Model (Exposure Contrast)	Adjusted Effect (95% CI)	*p* (Raw)	*p* (BH-FDR)
Length of stay, days	Patient-level linear regression (any GA exposure vs. SA–SA)	β = +0.20 (−0.68, +1.07)	0.661	0.661
Intra-operative MBP < 65 mmHg events	Surgery-level NB GEE (GA vs. SA per surgery)	aIRR 0.29 (0.10–0.87) ^1^	0.027	0.108
Peri-operative Hb drop, g/dL	Surgery-level linear mixed-effects (GA vs. SA per surgery)	β = −0.16 (−0.40, +0.09)	0.211	0.423
Peak post-operative log-CRP	Patient-level linear regression (any GA exposure vs. SA–SA)	β = +0.04 (−0.10, +0.18)	0.605	0.661

^1^ Adjusted incidence rate ratio (aIRR) from the negative-binomial model (log-scale coefficient −1.23; exp[−1.23] ≈ 0.29), corresponding to numerically fewer hypotensive events under GA at raw *p* (*p* = 0.027); this difference did not persist after BH-FDR correction (*p* = 0.108) and is not interpreted as a reliable effect. Models used the pre-specified Table 3 clinical covariate set; baseline laboratory adjustment was endpoint-specific—the length-of-stay and peak-CRP models included baseline hemoglobin and creatinine, the hemoglobin-drop model baseline hemoglobin, and the hypotension model baseline creatinine—with the length-of-stay model additionally adjusted for the inter-surgery interval and the peak-CRP model for baseline albumin. Patient-level models use across-surgery averages of tourniquet and operation times; surgery-level models use per-surgery values + surgery number. Complications reported descriptively (4/207, 1.9%)—see Section 2.4. Abbreviations: BH-FDR, Benjamini–Hochberg false discovery rate; NB, negative binomial; GEE, generalized estimating equations; MBP, mean blood pressure; Hb, hemoglobin; CRP, C-reactive protein.

The two co-primary endpoints were tested without multiple-comparison correction, given pre-specified hypotheses with separate rationales; the four-pattern and surgery-level AKI models were treated as exploratory, so the nominally significant GA–SA contrast (*p* = 0.025) carries no confirmatory status. The Benjamini–Hochberg false discovery rate (BH-FDR, α = 0.05) was applied within the secondary-endpoint family and, separately, within the AKI sensitivity family [32]; two-sided *p* < 0.05 was significant. Six sensitivity analyses were pre-specified—AT classification; the four-pattern analysis; an extended cohort re-including the six 14-day-interval patients; a stricter cohort excluding pre-Op1 eGFR below 60 mL/min/1.73 m^2^; exclusion of the 12 imputed-CRP patients; and a stricter AKI definition (KDIGO Stage 2 or higher)—and three post hoc analyses were added: 1:1 nearest-neighbor propensity-score matching (caliper 0.2 standard deviations of the logit propensity score; 51 pairs; cluster-robust standard errors) [33], inverse-probability-of-treatment weighting (stabilized, truncated weights), and the Op2-specific baseline AKI ascertainment, with the propensity and weighting models augmenting the covariate set with baseline hemoglobin. Covariate balance for the weighted analysis was assessed by standardized mean differences, propensity-score overlap, and the Kish effective sample size (Supplementary Table S5 and Figures S1 and S2). Analyses used Python 3.11.9 with pandas 3.0.2, NumPy 2.4.4, SciPy 1.17.1, and statsmodels 0.14.6; the analysis plan was pre-specified, post hoc additions are labeled, and analytic code is available on request.

## 3. Results

### 3.1. Cohort Characteristics

Of 216 patients screened, 9 were excluded, leaving 207 patients (414 surgeries) for the primary analysis (Figure 1). Baseline characteristics are summarized in Table 1; the cohort was predominantly female with a median age of 75 years. The realized inter-surgery interval was 7 days in 196 of 207 patients (94.7%; range 7–9), and the 6 patients with a 14-day interval were excluded from the primary cohort and re-included only in the extended-cohort sensitivity analysis. Under the as-treated framework, 306 surgeries (73.9%) were performed under SA and 108 (26.1%) under GA; 42 surgeries originally classified as GA documented an attempted spinal injection followed by intra-operative conversion (42 of 348 attempted spinal anesthetics, approximately 12%), and reassigning these to SA under the initial-anesthetic-plan (intention-to-treat) analysis yielded 348 SA (84.1%) and 66 GA (15.9%) surgeries. Anesthesia time was longer under GA than SA (Table 2), reflecting the additional induction, positioning, and emergence phases of general anesthesia rather than a longer operation, as operation time was comparable; tourniquet time, intake fluid, and mean and minimum MBP were also similar between groups (Table 2). Intra-operative vasopressor use was more frequent under spinal than general anesthesia (any ephedrine or phenylephrine, 37.4% versus 12.1% of surgeries; *p* < 0.001; Supplementary Table S7), whereas intra-operative fluid volumes did not differ.

### 3.2. Acute Kidney Injury

**Figure 2:**
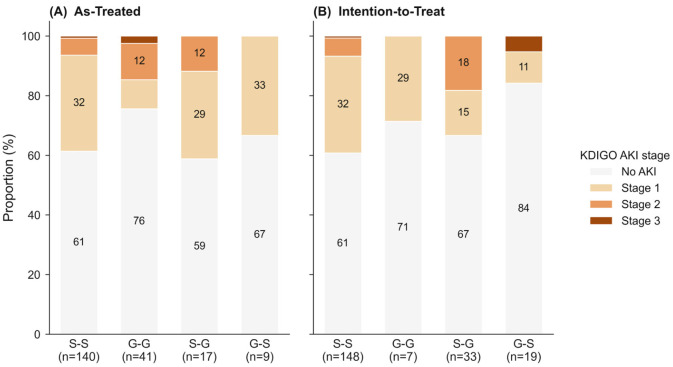
AKI stage distribution by anesthesia pattern. Patient-level worst KDIGO stage within each anesthesia pattern. (**A**) As-treated classifies each surgery by the anesthesia actually administered, with the 42 spinal-failure conversions counted as GA; (**B**) intention-to-treat, the primary framework, reclassifies these to SA. The same 207 patients appear in both panels; only the pattern labels change. Segment percentages shown when at least 10%.

**Figure 3:**
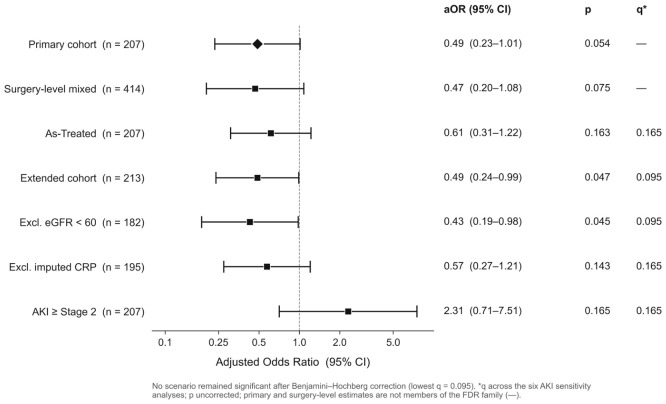
Adjusted odds ratio of AKI: primary and sensitivity analyses. Forest plot of adjusted odds ratios (95% CI) for any GA exposure versus SA–SA, or per-surgery GA in the surgery-level model. The diamond denotes the primary patient-level estimate and squares the sensitivity scenarios; the adjacent table lists the adjusted odds ratio (95% CI), the uncorrected *p*, and the Benjamini–Hochberg q for the six AKI sensitivity analyses. No scenario remained significant after correction (lowest q = 0.095); markers are shown without color emphasis so that the nominally significant scenarios are not over-stated.

Peri-operative AKI occurred in 74 of 207 patients (35.7%) at the patient level and in 94 of 414 surgeries (22.7%; Table 2), more often after the first surgery than the second (65 of 207, 31.4%, versus 29 of 207, 14.0%); among the 74 patients, 57 had Stage 1, 15 Stage 2, and 2 Stage 3 (Figure 2). Post-operative creatinine was essentially complete (at least one value within every protocol-defined window for ≥99% of patients overall; median eight measurements per patient), with no significant difference in availability by anesthesia group (lowest single-window subgroup availability 96.6%; all *p* ≥ 0.14; Supplementary Table S3). The adjusted patient-level estimate for any GA exposure versus SA–SA did not reach statistical significance (aOR 0.49, 95% CI 0.23–1.01, *p* = 0.054; Table 3). When restricted to KDIGO Stage 2 or higher (17 events), the estimate reversed direction (aOR 2.31, 95% CI 0.71–7.51, *p* = 0.165), although underpowered. In the four-pattern secondary model, the GA–SA pattern (n = 19) reached nominal significance versus SA–SA (aOR 0.21, 95% CI 0.05–0.82, raw *p* = 0.025) but did not survive BH-FDR correction (q = 0.095; Supplementary Table S1); the GA–GA and SA–GA patterns were non-significant (Table 3). The surgery-level model yielded aOR 0.47 (95% CI 0.20–1.08, *p* = 0.075) under intention-to-treat (as-treated estimates, Table 3; adjusted effects across analyses, Figure 3).

### 3.3. Transfusion

PRBC transfusion occurred in 185 of 414 surgeries (44.7%) and in 138 of 207 patients (66.7%; Table 1). After adjustment, the per-surgery probability of transfusion did not differ between SA and GA under either intention-to-treat (aOR 0.90, 95% CI 0.50–1.62, *p* = 0.728) or as-treated (aOR 1.13, 95% CI 0.72–1.77, *p* = 0.609; Table 3), and transfusion units per surgery and patient-level total units likewise did not differ (Table 3).

### 3.4. Secondary Endpoints and Complications

**Figure 4:**
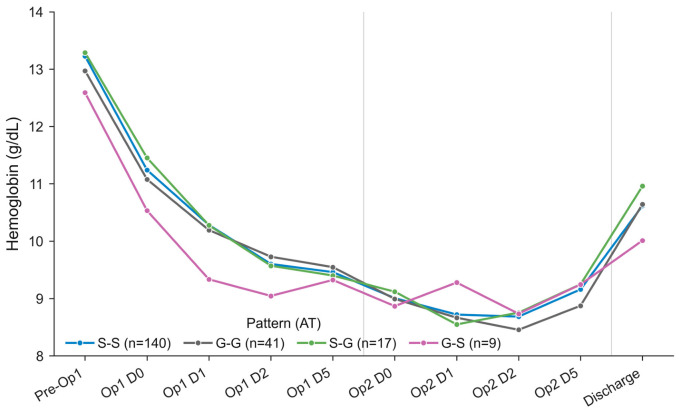
Hemoglobin trajectory by anesthesia pattern. Mean hemoglobin at ten peri-operative timepoints, stratified by as-treated pattern. Vertical lines separate the first-surgery, second-surgery, and discharge phases. Abbreviations: S, spinal anesthesia; G, general anesthesia; AT, as-treated.

Length of stay and peak post-operative CRP (patient-level, any GA exposure versus SA–SA) and peri-operative Hb drop (surgery-level, GA versus SA per surgery) showed no significant difference after adjustment (all BH-FDR *p* > 0.40; Table 4). Intra-operative MBP < 65 mmHg event counts were numerically lower under GA (adjusted incidence rate ratio [aIRR] 0.29, 95% CI 0.10–0.87, raw *p* = 0.027) but did not persist after BH-FDR correction (*p* = 0.108; Table 4). The hemoglobin decline was concentrated around the first surgery, whereas transfusion was more frequent around the second (Figure 4; Supplementary Table S6): the within-surgery local Hb drop was larger after the first surgery, patients entered the second surgery already anemic, and transfusion was more frequent at the second surgery (all values in Supplementary Table S6; both *p* < 0.001). The composite complication endpoint occurred in 4 of 207 patients (1.9%; Table 1); no patient developed hematoma, SSI, DVT, or PE, and 4 underwent reoperation.

### 3.5. Sensitivity Analyses

Across the pre-specified binary sensitivity scenarios (Supplementary Table S2, Figure 3), the point estimate remained below 1.0 except under the restricted KDIGO Stage 2-or-higher definition; the extended-cohort and stricter-renal-baseline scenarios reached nominal significance, whereas the exclude-imputed-CRP and Stage 2-or-higher scenarios did not. No scenario crossed q < 0.05 after BH-FDR correction across the six AKI sensitivity *p*-values (lowest q = 0.095; Supplementary Table S1). Propensity-score matching (51 pairs) and the Op2-specific baseline (under which the patient-level event count rose from 74 to 103) each kept the point estimate below 1.0 but remained non-significant, whereas inverse-probability-of-treatment weighting reached nominal significance (Supplementary Table S1); none of these post hoc analyses carries confirmatory status. Covariate balance improved substantially after weighting (maximum standardized mean difference 0.14 for hypertension, all others ≤0.09), with adequate overlap and a Kish effective sample size of 180 of 207 (87%), stable across weight truncation (Supplementary Table S5 and Figures S1 and S2). Firth penalized-likelihood re-estimation left the any-GA estimate essentially unchanged (aOR 0.52, 95% CI 0.26–1.07, *p* = 0.075), stable also with the propensity score as a single covariate (aOR 0.54) and under a reduced five-covariate model (aOR 0.58; Supplementary Table S4). The adjusted transfusion effect remained non-significant across all scenarios (Supplementary Table S2).

## 4. Discussion

### 4.1. Main Findings

In this cohort of 207 older Korean adults undergoing one-week-staged primary BTKA, anesthesia technique was not significantly associated with peri-operative AKI or transfusion. The adjusted patient-level odds ratio for any GA exposure versus SA–SA was 0.49 (95% CI 0.23–1.01, *p* = 0.054)—directionally opposite to our a priori hypothesis. Three of six pre-specified AKI sensitivity analyses crossed α = 0.05 at raw *p* (the four-pattern GA–SA contrast, *p* = 0.025; the extended cohort, *p* = 0.047; the stricter renal baseline, *p* = 0.045), yet none retained significance after BH-FDR correction. The primary estimate chiefly reflected mild injury (Stage 1, 57 of 74 events), and restriction to KDIGO Stage 2 or higher (17 events) reversed the direction (aOR 2.31, 95% CI 0.71–7.51, *p* = 0.165); this severity reversal renders the adjusted estimates directionally inconsistent and best regarded as hypothesis-generating, with both severity strata underpowered. Two propensity-based analyses agreed in direction with the primary estimate but differed in significance. Transfusion, length of stay, hemoglobin drop, and peak CRP showed no significant difference after adjustment, with confidence intervals not excluding clinically relevant differences either way—no evidence of difference rather than evidence of equivalence. The null transfusion result is plausible: transfusion after staged bilateral arthroplasty is likely dominated by cumulative blood loss and a uniform threshold rather than anesthetic technique, and the substantial burden in this cohort—which used no routine antifibrinolytic prophylaxis—did not differ between techniques. Consistent with this, the largest absolute hemoglobin decline occurred after the first surgery, and patients entered the second already anemic near the transfusion threshold (Section 3.4; Figure 4); the smaller measured second-surgery drop therefore reflects cumulative anemia and more frequent peri-Op2 transfusion, not less surgical bleeding at the second operation (direct blood-loss measures were unavailable). Intra-operative hypotensive events occurred numerically more often under SA, in a direction compatible with the known hemodynamic profile of spinal anesthesia in older adults [34], although the adjusted difference did not survive BH-FDR correction (Table 4), so this remains a numerical trend.

### 4.2. Comparison with Previous Studies

The contrast with prior reports favoring neuraxial anesthesia may reflect differences in population, surgical setting, and AKI definition. Our results partially align with the Korean single-center analysis of 2987 TKAs by Kim et al. [23], which found only a non-significant trend toward fewer AKI events under SA, whereas larger Korean propensity-matched [21] and nationwide [22] analyses favored regional anesthesia for several short-term outcomes across mostly unilateral procedures, unlike our older, predominantly female staged-bilateral cohort. A Korean machine-learning model identified GA as a contributing feature for post-operative AKI [24] but was developed for prediction rather than confounder-adjusted comparison. Asian data from Singapore favor neuraxial techniques for unilateral TKA [16], whereas Japanese [4] and Chinese [35] analyses compared simultaneous versus staged arthroplasty without contrasting anesthesia, and Korean cohorts did not isolate anesthesia type [2,10,36]. Large-database and pooled analyses consistently favor neuraxial anesthesia for unilateral TKA on transfusion, length of stay, readmission, morbidity, and discharge [14,15,17,18,19,20,37], and a recent National Surgical Quality Improvement Program analysis of patients aged ≥75 years reported a spinal-favorable early-recovery and discharge profile [38]. The Veterans Affairs cohort by Baldawi et al. reported lower renal complication risk under neuraxial anesthesia [25], contrasting with our trend, though that predominantly male, mostly unilateral population differs from ours. Any neuraxial transfusion advantage may be modest and setting-dependent; in this cohort—with uniform tourniquet use but no routine antifibrinolytic prophylaxis—none was detected despite a substantial transfusion burden, consistent with a four-arm trial in which tourniquet use, rather than anesthetic technique, independently reduced hemoglobin decline [26]. Finally, because the directional pattern was confined to mild Stage 1 events, binary AKI definitions may reach different conclusions depending on the severity distribution, consistent with bilateral-TKA reports using alternative non-KDIGO AKI definitions [7] and the documented amplification of AKI risk in bilateral procedures [5,6] and lower-limb arthroplasty [39].

### 4.3. Clinical and Methodological Implications

Our findings should not be read as evidence that GA is superior to SA in older adults undergoing staged BTKA; rather, the spinal-favorable associations reported in unilateral TKA may not extend uniformly to this setting. The anesthesia decision should remain individualized, weighing risk factors—including chronic kidney disease, baseline BMI, hemodynamic tolerance, and spinal anatomy relevant to neuraxial access [40]—contraindications, cost and resource considerations [41], and patient preference. A recent randomized trial in frail older patients found that differing intra-operative mean-blood-pressure targets (65–85 versus 85–100 mmHg) did not alter early recovery or AKI [42], suggesting that mechanisms beyond intra-operative blood pressure—such as sympathetic blockade and the surgical stress response—may be more relevant. The 42 conversions carry analytic weight, because observational studies rarely state whether conversions are handled under intention-to-treat or as-treated; as-treated classification attenuated the AKI signal (aOR 0.61, *p* = 0.163), reflecting the baseline risk profile of patients initially selected for SA. A conversion rate of this magnitude in an older, predominantly female cohort is noteworthy—degenerative and osteoporotic spinal changes may reduce neuraxial reliability—although its drivers were not characterized here; we recommend pre-specifying conversion handling with chart-review validation and dedicated predictor analysis. Because selection was non-random and the spinal-to-general ratio was imbalanced (approximately 3:1 under as-treated), propensity-score matching (51 pairs; aOR 0.65, 95% CI 0.25–1.72, *p* = 0.386) and inverse-probability-of-treatment weighting (aOR 0.40, 95% CI 0.20–0.81, *p* = 0.011)—both post hoc analyses lying outside the six-member pre-specified AKI sensitivity family and reported with raw *p*-values only—agreed in direction with the primary result but differed in significance, and are best read as a non-confirmatory sensitivity range. We now report weighting diagnostics (balance, overlap, and effective sample size; Supplementary Table S5 and Figures S1 and S2), which are reassuring, but the weighted estimate remains susceptible to residual confounding and should not be privileged over the primary multivariable estimate. Finally, because Op1 and Op2 were only seven days apart, an Op2-specific baseline rather than the single pre-Op1 baseline aligned directionally with the primary analysis (aOR 0.62, 95% CI 0.32–1.20, *p* = 0.156) but raised the event count from 74 to 103, so the single baseline materially under-counts new post-Op2 injury. We nonetheless retained the single pre-Op1 baseline as the pre-specified primary because it provides one uniform pre-operative reference shared by both surgeries and is the conservative choice—any resulting misclassification, if non-differential, biases the exposure estimate toward the null—and the adjusted association was stable across baseline definitions (aOR 0.49 versus 0.62), so the baseline choice shifts the event count more than the exposure estimate. Dual-baseline reporting nonetheless warrants standardization in future staged-bilateral AKI studies.

### 4.4. Strengths and Limitations

Strengths include a uniformly defined cohort under a consistent single-institution surgical protocol, modeling of within-patient correlation with mixed-effects regression, current KDIGO creatinine-based AKI definitions with a pre-Op1 baseline for all patients, and six pre-specified sensitivity analyses. Comparative data specific to the one-week-staged bilateral setting remain scarce—the principal gap this analysis addresses—although the single-center scope limits external validity and motivates multi-center replication.

Several limitations apply. First, the retrospective single-center design carries selection bias and confounding by indication whose direction is not neutral: clinicians may preferentially select GA for frailer or higher-risk patients—the GA group had higher chronic-kidney-disease prevalence (6.1% versus 1.1%, Table 2), an imbalance that alone would bias the GA estimate toward harm rather than benefit; conversely, spinal failure may itself cluster in higher-risk patients, leaving the net direction of confounding uncertain, so the lower AKI odds under GA must not be read as renoprotection; surgeon identity and year of surgery (January 2020–April 2026) were not modeled, leaving residual confounding from practice drift. Intra-operative vasopressor use (more frequent under spinal anesthesia) and intra-operative fluid volumes were captured (Supplementary Table S7) but were not entered into the primary adjustment set and thus remain potential residual confounders; frailty, functional status, peri-operative fluid balance beyond the recorded intra-operative volume, and post-operative nephrotoxic-medication exposure were unavailable and remain unmeasured. Second, with no a priori power calculation, the analyses are exploratory; samples for severe AKI (17 events) and complications (4 events) are small, and the patient-level AKI model fit 14 coefficients on 74 events (events-per-variable ≈ 5, below the target of 10); the borderline estimate, however, was confirmed by Firth penalized-likelihood, reduced-model, and propensity-score-as-covariate refits (Supplementary Table S4) not to be an artifact of the events-per-variable ratio, and the nominally significant GA–SA pattern (n = 19) is additionally confounded with surgery order. Third, the 42 conversions were identified by an automated text-and-dosing query and confirmed by the operating anesthesiologist as genuine spinal-anesthesia failures, although a formal blinded per-case chart audit by an independent assessor was not performed. Fourth, propensity analyses [33] cannot exclude unmeasured confounding; the reported overlap, balance, and effective-sample-size diagnostics (Supplementary Table S5 and Figures S1 and S2) were reassuring, but every non-null estimate—including the significant weighted result—is interpreted as compatible with residual confounding. Fifth, the complication composite (4 of 207, 1.9%) is descriptive only. Sixth, AKI ascertainment used creatinine alone (urine-output data were not consistently documented) against a single pre-Op1 baseline—a recognized limitation in staged-bilateral cohorts that motivates the baseline standardization discussed above. Seventh, the co-primary endpoints were tested without between-primary correction; under a Bonferroni threshold (α = 0.025), neither AKI (*p* = 0.054) nor transfusion (*p* = 0.728) would be significant, reinforcing the null interpretation. Eighth, the transfusion analysis carries specific limitations: direct intra-operative blood-loss measures (estimated blood loss, drain output) were not recorded, so the comparison cannot be anchored to actual surgical bleeding; transfusion was not governed by a rigid protocol but by a hemoglobin threshold below 8 g/dL or symptomatic anemia, so clinician judgment may have contributed to the high transfusion rate even though the group-mean hemoglobin trajectory (Figure 4) generally remained above the 8 g/dL transfusion threshold, whereas individual nadirs fell lower; because the two surgeries were only about one week apart, both second-surgery transfusion and the pre-Op1-referenced hemoglobin drop partly reflect cumulative first-surgery losses, which we accommodated through within-patient clustering and by reporting the within-surgery local hemoglobin drop (Supplementary Table S6) but could not fully separate. Finally, the cohort was exclusively older Korean patients undergoing one-week-staged BTKA, so generalization to other populations, intervals, or simultaneous BTKA should be cautious [43].

### 4.5. Future Directions

These observations should not modify current practice but underscore the need for prospective multi-center replication adequately powered for severe AKI (KDIGO Stage 2 or higher), with severity-stratified reporting and explicit pre-specification of conversion handling. Future trials should also characterize intra-operative hemodynamic targets, given evidence that broad mean-blood-pressure targets did not themselves alter early recovery or AKI in frail older adults [42], and better characterize spinal-anesthesia failure—its predictors, rate across institutions, and impact on outcomes.

## 5. Conclusions

In this single-center cohort of 207 older Korean adults undergoing one-week-staged primary bilateral total knee arthroplasty, anesthesia technique was not significantly associated with peri-operative AKI or transfusion. The primary patient-level adjusted AKI estimate did not reach significance (*p* = 0.054); three sensitivity-analysis *p*-values crossed α = 0.05 at raw p but none retained significance after BH-FDR correction, restriction to KDIGO Stage 2 or higher reversed the direction, and the two propensity-based estimates agreed in direction but differed in significance, so—together with an events-per-variable ratio of approximately 5—we regard these findings as hypothesis-generating rather than confirmatory. The lower AKI odds under GA reflected predominantly mild Stage 1 events and were underpowered, especially for severe AKI (17 events). Transfusion—the second co-primary endpoint—together with length of stay, hemoglobin drop, peak CRP, and intra-operative hypotension, likewise showed no significant difference between techniques after multiplicity correction, so our a priori hypothesis that SA would be protective was not confirmed for either. Approximately 12% of attempted spinal anesthetics converted intra-operatively to GA, and the analytic handling of these conversions influenced the observed effect. These observations do not support modifying current practice but emphasize the need for prospective multi-center replication adequately powered for severe AKI, with severity-stratified reporting and explicit pre-specification of how conversions are analyzed.

## Data Availability

The de-identified analytic dataset that supports the findings of this study is available from the corresponding author on reasonable request and subject to renewed institutional data-use approval. The raw electronic medical record data are not publicly available because they contain protected health information, and their public release is restricted by the approving Institutional Review Board and by Korean medical-information protection regulations. The Python analysis code used to derive the analytic cohort, fit the statistical models, and generate the figures is likewise available from the corresponding author on reasonable request.

## Supplementary Materials

The following supporting information can be downloaded at https://www.mdpi.com/article/10.3390/jcm15134937/s1. Table S1: Pre-specified AKI sensitivity family (with BH-FDR) and post-hoc sensitivity analyses for the AKI primary endpoint; Table S2: Sensitivity Analyses for Primary Endpoints (Adjusted Effects of GA Exposure); Table S3: Completeness of post-operative serum creatinine, overall and by anesthesia group; Table S4: Robustness of the patient-level any-general-anesthesia AKI estimate to penalized and reduced-model estimation; Table S5: Propensity-score covariate balance, overlap, and effective sample size for the inverse-probability-of-treatment-weighted analysis; Table S6: First- versus second-surgery hemoglobin decline, transfusion, and acute kidney injury; Table S7: Intra-operative vasopressor use, fluid administration, and urine output by anesthesia group; Figure S1: Standardized mean differences before and after inverse-probability-of-treatment weighting (Love plot); Figure S2: Propensity-score overlap (common support); STROBE checklist.
