# Supplementary material for: Spinal Versus General Anesthesia for Acute Kidney Injury and Transfusion in One-Week-Staged Bilateral Total Knee Arthroplasty
Source: J Clin Med. 2026 Jun 25;15(13):4937. doi: 10.3390/jcm15134937 (PMC13361103; doi:10.3390/jcm15134937)
Supplement: Supplementary file 1 [file jcm-15-04937-s001.zip › Table_S1_REV1_260618.pdf]

Table S1. Pre-specified AKI sensitivity family (with BH-FDR) and post-hoc sensitivity analyses for the AKI primary endpoint

Analysis	Comparison	aOR	95% CI	*p*	BH-FDR *q*	N (events)
**Pre-specified AKI sensitivity family** (BH-FDR applied)						
As-treated classification	Any GA exposure vs SA–SA	0.61	0.31–1.22	0.163	0.165	207 (74)
4-pattern analysis (GA–SA vs SA–SA)	GA–SA pattern vs SA–SA	0.21	0.05–0.82	0.025	0.095	207 (74)
Extended cohort (+ 6 patients, 14-day interval)	Any GA exposure vs SA–SA	0.49	0.24–0.99	0.047	0.095	213 (event count not reported)[1]
Stricter renal baseline (eGFR ≥ 60)	Any GA exposure vs SA–SA	0.43	0.19–0.98	0.045	0.095	182 (event count not reported)[1]
Exclude imputed post-Op2 CRP (12 within cohort, 1 already excluded)	Any GA exposure vs SA–SA	0.57	0.27–1.21	0.143	0.165	195 (event count not reported)[1]
KDIGO Stage ≥ 2 only	Any GA exposure vs SA–SA	2.31	0.71–7.51	0.165	0.165	207 (17)
**Post-hoc sensitivity analyses**						
Propensity-score matching (1:1, caliper 0.2 × SD of logit PS, cluster-robust SE by pair)	Any GA exposure vs SA–SA	0.65	0.25–1.72	0.386	—	51 matched pairs
Inverse-probability-of-treatment weighting (stabilized, 1–99% trunc.)	Any GA exposure vs SA–SA	0.40	0.20–0.81	0.011	—	207
Op2-specific baseline (latest pre-Op2 Cr by lab date; POD5 in 200/207, POD2 in 7/207)	Any GA exposure vs SA–SA	0.62	0.32–1.20	0.156	—	207 (103)

[1] AKI event count was not separately tabulated for the alternate cohort definitions; patient-count N is shown for the cohort. The BH-FDR family is restricted to the six pre-specified AKI sensitivity analyses (one BH adjustment applied to all six contrasts entered in this table's upper block). For the 4-pattern model, the GA–SA-versus-SA–SA per-pattern contrast (aOR 0.21, $p = 0.025$) was the single member entered into the BH-FDR family (yielding $q = 0.095$); the remaining per-pattern contrasts (GA–GA, SA–GA) are reported descriptively in Table 3 and were not entered into the family.

aOR, adjusted odds ratio; CI, confidence interval; BH-FDR, Benjamini–Hochberg false discovery rate; AKI, acute kidney injury; KDIGO, Kidney Disease: Improving Global Outcomes; SA, spinal anesthesia; GA, general anesthesia; eGFR, estimated glomerular filtration rate; CRP, C-reactive protein; Cr, creatinine; POD, post-operative day; IPTW, inverse-probability-of-treatment weighting; PS, propensity score; SE, standard error. BH-FDR q*-values are computed across the six pre-specified AKI sensitivity analyses only; the three post-*

hoc analyses are reported with raw p*-values. The six pre-specified AKI sensitivity analyses use the same adjustment set as the primary patient-level AKI model: age, sex, BMI, ASA class ≥ 3, hypertension, diabetes mellitus, cardiovascular disease, chronic kidney disease, antiplatelet and anticoagulant use, baseline creatinine, mean tourniquet time (average of Op1 and Op2), and mean surgical time (average of Op1 and Op2). The three post-hoc analyses (propensity-score matching, IPTW, and the Op2-specific baseline) used this set with the addition of baseline hemoglobin in the propensity/adjustment model.*
