# Supplementary material for: Spinal Versus General Anesthesia for Acute Kidney Injury and Transfusion in One-Week-Staged Bilateral Total Knee Arthroplasty
Source: J Clin Med. 2026 Jun 25;15(13):4937. doi: 10.3390/jcm15134937 (PMC13361103; doi:10.3390/jcm15134937)
Supplement: Supplementary file 1 [file jcm-15-04937-s001.zip › Table_S2_REV1_260618.pdf]

Table S2. Sensitivity Analyses for Primary Endpoints (Adjusted Effects of GA Exposure)

#	Scenario	N (patients / surgeries)	AKI: aOR (95% CI), *p*	Transfusion occurrence: aOR (95% CI), *p*
S0	**Primary** (ITT, primary cohort)	207 / 414	0.49 (0.23–1.01), 0.054	0.90 (0.50–1.62), 0.728
①	As-Treated (sensitivity exposure)	207 / 414	0.61 (0.31–1.22), 0.163	1.13 (0.72–1.77), 0.609
③	Extended cohort (re-include 14-day interval)	213 / 420 ‡	**0.49 (0.24–0.99), 0.047**	0.77 (0.43–1.37), 0.370
④	Stricter cohort (exclude eGFR < 60)	182 / 364	**0.43 (0.19–0.98), 0.045**	1.15 (0.61–2.18), 0.661
⑤	Exclude imputed-CRP patients within cohort (n=12; 1 of 13 already excluded)	195 / 390	0.57 (0.27–1.21), 0.143	0.89 (0.48–1.62), 0.693
⑥	Restricted AKI definition (KDIGO ≥ Stage 2 only)	207	2.31 (0.71–7.51), 0.165*	n/a

Adjusted effect of any GA exposure on patient-level AKI and of surgery-level GA exposure on transfusion. Same covariates as Table 3. Bold $p < 0.05$.

Both the ITT (0.90) and as-treated (1.13) transfusion-occurrence estimates are non-significant with wide, unity-spanning confidence intervals; the apparent change in point-estimate direction between the two exposure definitions is therefore not interpretable.

Scenario ② (4-pattern analysis) is not tabulated here because each row reports a single binary any-GA-versus-SA–SA contrast; the per-pattern 4-pattern estimates are in Table 3(A) and the 4-pattern contrast with BH-FDR correction is in Table S1.

* Stage ≥ 2 includes only 17 events (7 in any-GA-exposure patterns, 10 in SA–SA, under intention-to-treat); exploratory.

‡ The 6 re-included 14-day-interval patients contributed 213 patients to the patient-level AKI model; anesthesia-record data were available for only 6 of their 12 surgeries, so the surgery-level transfusion model used 420 surgeries (414 primary + 6). The patient-level AKI estimate (bold) is unaffected.

Abbreviations: aOR, adjusted odds ratio; CI, confidence interval; ITT, intention-to-treat (initial anesthetic plan); SA, spinal anesthesia; GA, general anesthesia; AKI, acute kidney injury; KDIGO, Kidney Disease: Improving Global Outcomes.
