# Supplementary material for: Spinal Versus General Anesthesia for Acute Kidney Injury and Transfusion in One-Week-Staged Bilateral Total Knee Arthroplasty
Source: J Clin Med. 2026 Jun 25;15(13):4937. doi: 10.3390/jcm15134937 (PMC13361103; doi:10.3390/jcm15134937)
Supplement: Supplementary file 1 [file jcm-15-04937-s001.zip › Table_S3_REV1_260618.pdf]

Table S3. Completeness of post-operative serum creatinine, overall and by anesthesia group.

Proportion of the 207 patients with at least one serum creatinine value available within each protocol-defined post-operative window. *p*-values compare availability between the any-general-anesthesia and spinal–spinal groups (χ^2 test). Anesthesia groups defined under the initial-anesthetic-plan (intention-to-treat) framework. POD, post-operative day.

Window	Patients with creatinine, n	Overall, %	Any-GA, %	Spinal–spinal, %	*p*
Op1 POD0	207	100.0	100.0	100.0	1.00
Op1 POD1	205	99.0	96.6	100.0	0.14
Op1 POD2	207	100.0	100.0	100.0	1.00
Op1 POD5	207	100.0	100.0	100.0	1.00
Op2 POD0	207	100.0	100.0	100.0	1.00
Op2 POD1	205	99.0	96.6	100.0	0.14
Op2 POD2	207	100.0	100.0	100.0	1.00
Op2 POD5	206	99.5	100.0	99.3	1.00

Median number of post-operative creatinine measurements per patient, 8 (interquartile range 8–8); every patient had at least one post-operative creatinine value within both the first- and second-surgery windows (207/207, 100%). Availability did not differ by anesthesia group at any window.
