# Supplementary material for: Spinal Versus General Anesthesia for Acute Kidney Injury and Transfusion in One-Week-Staged Bilateral Total Knee Arthroplasty
Source: J Clin Med. 2026 Jun 25;15(13):4937. doi: 10.3390/jcm15134937 (PMC13361103; doi:10.3390/jcm15134937)
Supplement: Supplementary file 1 [file jcm-15-04937-s001.zip › Table_S4_REV1_260618.pdf]

Table S4. Robustness of the patient-level any-general-anesthesia AKI estimate to penalized and reduced-model estimation.

All models compare any general-anesthesia exposure with the spinal–spinal pattern for patient-level acute kidney injury (74 events / 207 patients). The maximum-likelihood full model is the primary analysis. Firth penalized-likelihood estimation addresses the approximately 5:1 events-per-variable ratio; the propensity-score-as-covariate and reduced specifications increase the events-per-variable ratio. aOR, adjusted odds ratio; CI, confidence interval; EPV, events per variable.

Specification	aOR	95% CI	*p*
Maximum likelihood — full (13 covariates + exposure = 14 coefficients; EPV ≈ 5; primary)	0.49	0.23–1.01	0.054
Firth penalized likelihood — full	0.52	0.26–1.07	0.075
Propensity score as a single covariate (EPV ≈ 37)	0.54	0.27–1.09	0.086
Reduced model (5 covariates)	0.58	0.30–1.15	0.120

The estimate was stable in direction and magnitude across all specifications and remained non-significant. For the exploratory four-pattern model, the small (3-event) GA–SA contrast shrank toward the null under Firth penalization (aOR 0.21 → 0.26; *p* 0.025 → 0.041), consistent with its labeling as exploratory and small-sample-fragile. Firth confidence intervals are penalized-Wald.
