# Supplementary material for: Spinal Versus General Anesthesia for Acute Kidney Injury and Transfusion in One-Week-Staged Bilateral Total Knee Arthroplasty
Source: J Clin Med. 2026 Jun 25;15(13):4937. doi: 10.3390/jcm15134937 (PMC13361103; doi:10.3390/jcm15134937)
Supplement: Supplementary file 1 [file jcm-15-04937-s001.zip › Table_S5_REV1_260618.pdf]

Table S5. Propensity-score covariate balance, overlap, and effective sample size for the inverse-probability-of-treatment-weighted analysis.

Absolute standardized mean differences (|SMD|) between the any-general-anesthesia and spinal–spinal groups before and after stabilized inverse-probability-of-treatment weighting (truncated at the 1st and 99th percentiles). |SMD| ≤ 0.10 indicates adequate balance. The propensity model used the pre-specified covariate set augmented with baseline hemoglobin. SMD, standardized mean difference.

Covariate	Absolute SMD, unweighted	Absolute SMD, after weighting
Age	0.30	0.02
Sex	0.17	0.04
Body mass index	0.41	0.09
ASA physical status ≥ 3	0.03	0.09
Hypertension	0.17	0.14
Diabetes mellitus	0.09	0.04
Cardiovascular disease	0.04	0.02
Chronic kidney disease	0.27	0.00
Antiplatelet use	0.00	0.02
Anticoagulant use	0.00	0.04
Baseline creatinine	0.14	0.08
Baseline hemoglobin	0.17	0.01
Tourniquet time	0.23	0.01
Operation time	0.13	0.00

After weighting, all standardized mean differences were ≤ 0.14, the maximum being 0.14 for hypertension and all others ≤ 0.09 (see also Supplementary Figure S1). Propensity-score overlap was adequate, with common support across the full range (any-GA 0.09–0.79; spinal–spinal 0.07–0.76) and no off-support observations (Supplementary Figure S2). The Kish effective sample size under weighting was 180 of 207 (87%), with a maximum stabilized weight of 2.99. The weighted estimate was stable across weight truncation (adjusted odds ratio 0.40 untruncated, 0.44 at the 5th–95th percentiles, and 0.40 at the 1st–99th percentiles), indicating it was not driven by extreme weights.
