# Supplementary material for: Spinal Versus General Anesthesia for Acute Kidney Injury and Transfusion in One-Week-Staged Bilateral Total Knee Arthroplasty
Source: J Clin Med. 2026 Jun 25;15(13):4937. doi: 10.3390/jcm15134937 (PMC13361103; doi:10.3390/jcm15134937)
Supplement: Supplementary file 1 [file jcm-15-04937-s001.zip › Table_S6_REV1_260618.pdf]

Table S6. First- versus second-surgery hemoglobin decline, transfusion, and acute kidney injury.

Within-patient comparison of the first (Op1) and second (Op2) staged surgeries (n = 207 patients). The local hemoglobin (Hb) drop is the fall from each surgery's pre-operative Hb to its post-operative nadir; the pre-Op2 Hb is the value on the morning of the second surgery. *p*-values are from the Wilcoxon signed-rank test (continuous) or McNemar exact test (transfusion occurrence). PRBC, packed red blood cells; IQR, interquartile range.

Variable	First surgery (Op1)	Second surgery (Op2)	*p*
Pre-operative Hb, g/dL, median	13.1	9.0	—
Local Hb drop, g/dL, median [IQR]	4.1 [3.4–4.7]	0.9 [0.3–1.3]	< 0.001
Transfused, n (%)	62 (30.0)	123 (59.4)	< 0.001
PRBC units, median (IQR)	0 (0–1)	1 (0–2)	< 0.001
Per-surgery AKI, n (%)	65 (31.4)	29 (14.0)	—

The large absolute hemoglobin decline occurred after the first surgery, from a near-normal baseline; patients entered the second surgery already anemic and close to the transfusion threshold (median interval Hb recovery ≈ 0 g/dL). The smaller measured Hb drop at the second surgery, despite more frequent peri-Op2 transfusion, reflects cumulative anemia and the floor imposed by the transfusion threshold rather than less surgical bleeding. This pattern corresponds to the hemoglobin trajectory in Figure 4.
