# Supplementary material for: Spinal Versus General Anesthesia for Acute Kidney Injury and Transfusion in One-Week-Staged Bilateral Total Knee Arthroplasty
Source: J Clin Med. 2026 Jun 25;15(13):4937. doi: 10.3390/jcm15134937 (PMC13361103; doi:10.3390/jcm15134937)
Supplement: Supplementary file 1 [file jcm-15-04937-s001.zip › Table_S7_REV1_260618.pdf]

Table S7. Intra-operative vasopressor use, fluid administration, and urine output by anesthesia group.

Surgery-level comparison under the initial-anesthetic-plan (intention-to-treat) framework (spinal, n = 348; general, n = 66). Vasopressor use is the proportion of surgeries receiving any ephedrine or phenylephrine; *p*-values are from the χ^2 test (proportions) or Mann–Whitney U test (volumes). IQR, interquartile range.

Variable	Spinal (n = 348)	General (n = 66)	*p*
Any vasopressor (ephedrine or phenylephrine), n (%)	130 (37.4)	8 (12.1)	< 0.001
Ephedrine administered, n (%)	111 (31.9)	8 (12.1)	0.002
Phenylephrine administered, n (%)	36 (10.3)	2 (3.0)	0.10
Intra-operative fluid, mL, median [IQR]	950 [800–1200]	1075 [900–1125]	0.40
Intra-operative urine output, mL, median (IQR)	0 (0–0)	0 (0–0)	0.62

Vasopressor administration was more frequent under spinal anesthesia, consistent with the recognized hemodynamic profile of spinal anesthesia in older adults. Intra-operative fluid volumes did not differ between groups. Hourly urine output was not reliably recorded in the retrospective electronic medical record and was therefore not used for KDIGO staging.
