# Supplementary figures and images for: Spinal Versus General Anesthesia for Acute Kidney Injury and Transfusion in One-Week-Staged Bilateral Total Knee Arthroplasty
Source: J Clin Med. 2026 Jun 25;15(13):4937. doi: 10.3390/jcm15134937 (PMC13361103; doi:10.3390/jcm15134937)

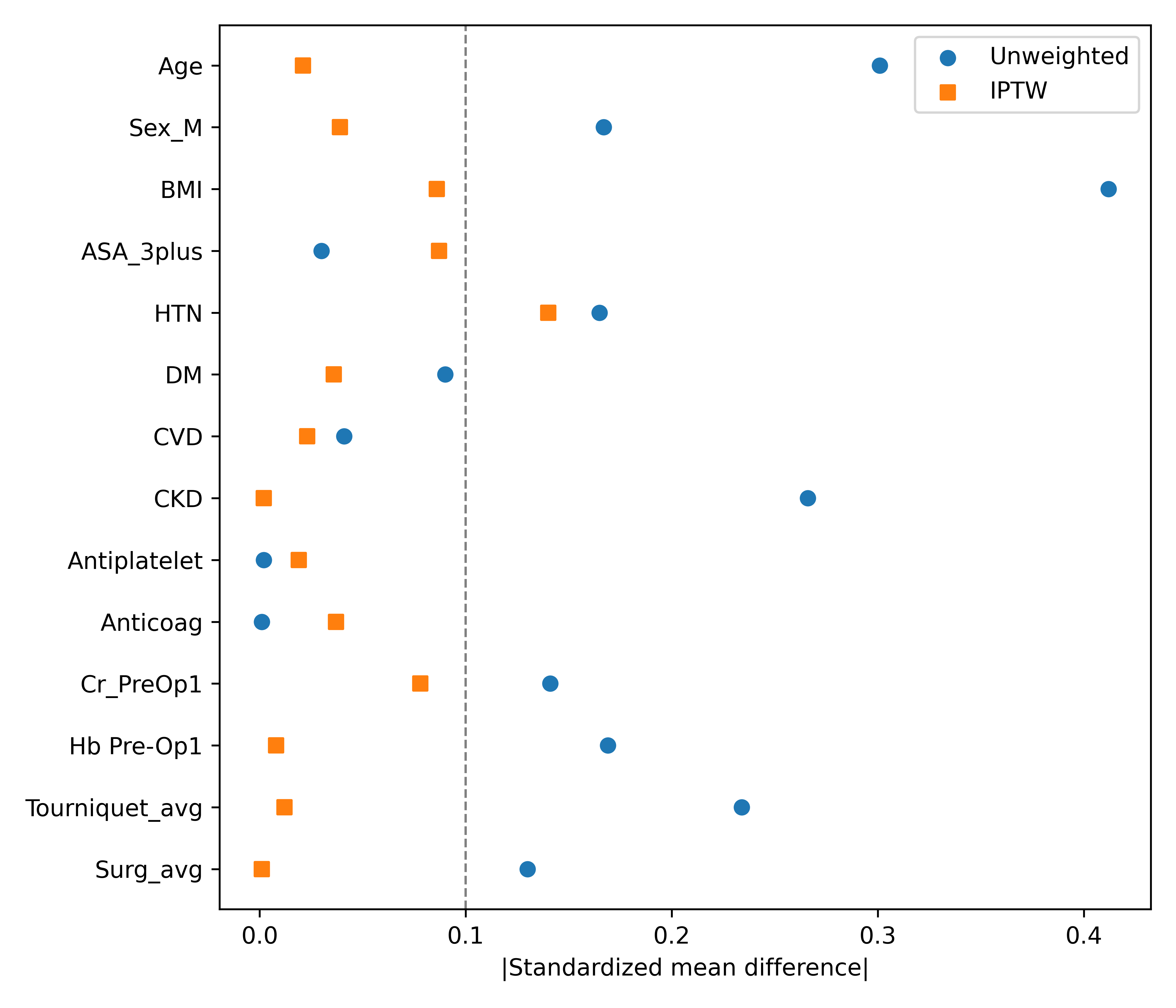

Supplement: Supplementary file 1 [file jcm-15-04937-s001.zip › fig_S1_love_plot.png]

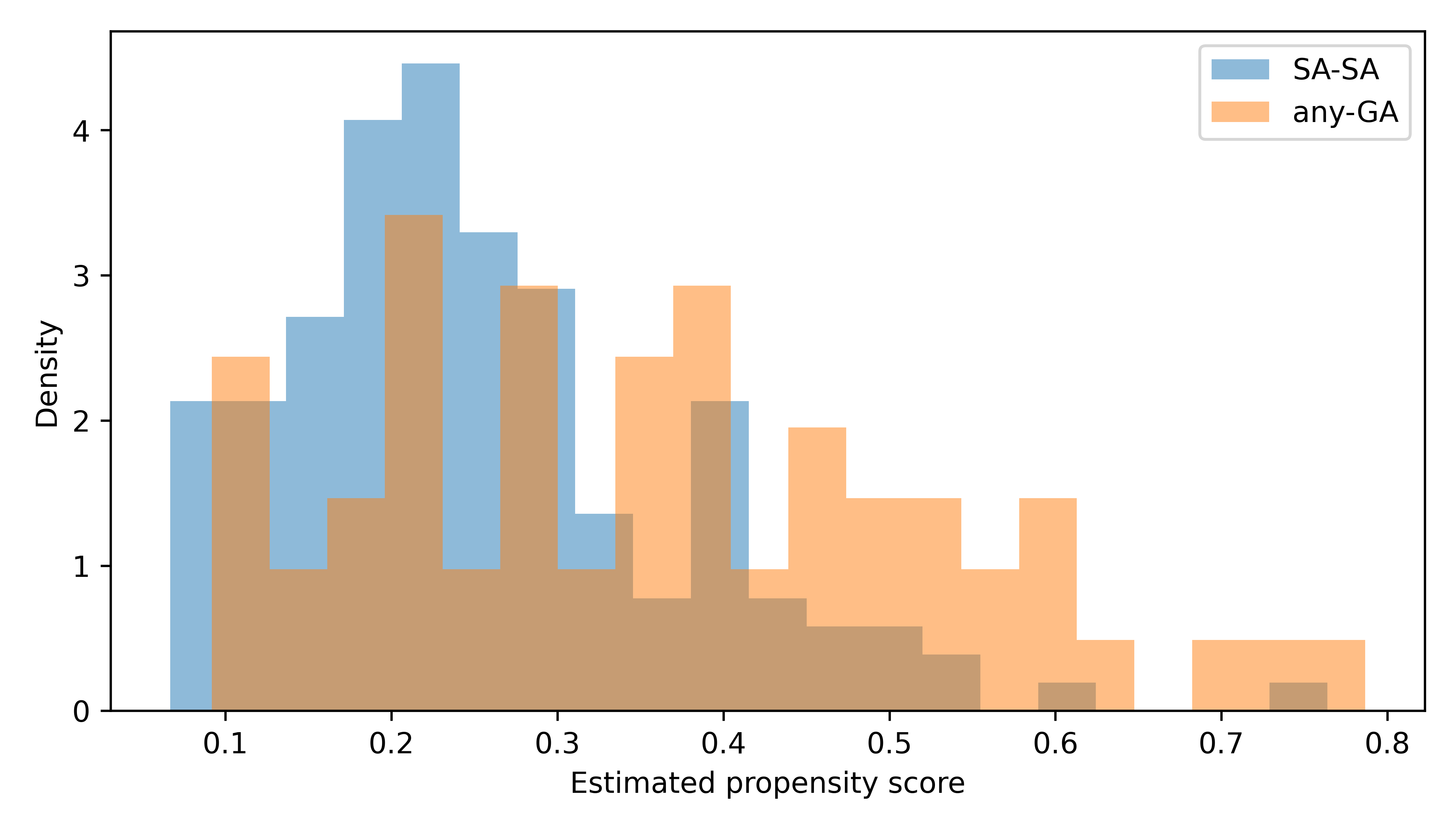

Supplement: Supplementary file 1 [file jcm-15-04937-s001.zip › fig_S2_ps_overlap.png]
